# Parental involvement of immigrant parents in early educational centers and its relationship with intercultural sensitivity

**DOI:** 10.3389/fpsyg.2025.1568532

**Published:** 2025-05-01

**Authors:** Marian Bilbao, Florencia Guglielmetti-Serrano, María José Mera-Lemp, José J. Pizarro, Denisse Fernandez, Gonzalo Martínez-Zelaya

**Affiliations:** ^1^Facultad de Psicología, Universidad Alberto Hurtado, Santiago, Chile; ^2^Escuela de Psicología, Universidad Gabriela Mistral, Santiago, Chile; ^3^Escuela de Ciencias Jurídicas y Sociales, Universidad Viña del Mar, Viña del Mar, Chile; ^4^Department of Social Psychology, University of the Basque Country UPV/EHU, Donostia-San Sebastián, Spain; ^5^Escuela de Psicología, Universidad Católica del Norte, Antofagasta, Chile

**Keywords:** immigrant parents, parental involvement, intercultural sensitivity, immigrant early education, intercultural education

## Abstract

**Introduction:**

Early education centers play a crucial role in children’s development. Research shows this process is partly supported by parental involvement in activities promoted by these centers. Additionally, early education helps reduce disparities and fosters inclusion of disadvantaged groups, such as immigrant families. These families often face barriers for their involvement, including limited understanding of the local culture, and teachers’ awareness of their cultural backgrounds and expectations. Literature suggests that interculturally sensitive practices addressing families’ cultural and linguistic needs can foster more effective communication between parents and teachers. Intercultural sensitivity, the affective dimension of intercultural communication, is a personal capacity enabling individuals to recognize and accept cultural differences while identifying commonalities. Thus, enhancing intercultural sensitivity within the educational community may support greater immigrant parental involvement in their children’s education. This study examines the role of intercultural sensitivity in predicting immigrant parental involvement in early education in Chile.

**Methods:**

Using a sample of 347 immigrant parents, we assessed parental involvement levels, intercultural sensitivity, and perceived facilitators and barriers to participation, with several sociodemographic variables.

**Results:**

Results from multinomial logistic regression showed that higher intercultural sensitivity significantly predicted greater parental involvement in children’s educational activities. Parents with lower intercultural sensitivity were 75% more likely to report “almost never” participating than those with high intercultural sensitivity. The educational level also played a role, as parents with technical education were significantly less involved than those with university education. Among facilitators, a positive climate among parents increased the odds of always participating, while the presence of other immigrant families in school paradoxically correlated with lower involvement. Regarding barriers, parents who perceived that the school lacked a special approach for immigrant families were 3.79 times more likely to report low participation.

**Discussion:**

These findings highlight the importance of fostering intercultural sensitivity in school communities to enhance immigrant parental engagement in early education. Implications for educational policy and practices promoting inclusive and culturally responsive environments are discussed.

## 1 Introduction

Infants’ development is particularly influenced by two environments: their home and the early education center (Halgunseth et al., 2009). In this regard, the relationship between families and their teachers is crucial, as the family is the first environment in which the child grows up and learns (Barahona Cruz et al., 2023; Keles et al., 2024). The study of parental involvement with early education centers has revealed that this participation supports children’s learning processes, establishes positive relationships with teachers and peers, develops socio-emotional tools, and acquires skills necessary for the transition into school (Barahona Cruz et al., 2023; Kang et al., 2024; Şahin et al., 2013; Sylva et al., 2004). In this context, the actions of parents and their role in their children’s early education are crucial, ranging from their involvement in the classroom (Rolla and Rivadeneira, 2006) to the home-based practices in which they participate (Kang et al., 2024; Kim and Yu, 2024).

Early education constitutes a fundamental educational space for children, providing the tools and stimulation necessary for adequate cognitive, emotional, and social development. Literature has shown that high-quality early education has positive effects on emotional self-regulation, preschool preparation, language development, understanding of the world, school success, and social competencies in both the short and long term (Barahona Cruz et al., 2023; Cebolla Boado, 2008; Kalmijn, 2024; Kang et al., 2024; Pinto and Misas, 2014; Rolla and Rivadeneira, 2006; Romero Díaz, 2024; Vargas Fonseca, 2024; Yamamoto and Li, 2012; Yoshikawa et al., 2013). Furthermore, early education also presents an opportunity to close socioeconomic gaps, reduce school dropout rates and juvenile crime, and promote the social inclusion of disadvantaged groups (Barnett and Frede, 2010; Hughes, 2022; Keles et al., 2024; Otero Bracco and Eisenberg, 2017).

Thus, a study in socioeconomically disadvantaged communities showed that parental involvement in early childhood education is important for performance in kindergarten and throughout the subsequent years of primary education, generating student motivation, positively impacting their academic performance, and, in turn, encouraging parents to remain engaged in their children’s education (Hayakawa et al., 2013). On the other hand, a study conducted with Latino immigrant parents in the United States has demonstrated the importance of early education centers, according to parents, for the socialization of their children and their integration into the community (Sánchez-Suzuki Colegrove, 2022), highlighting that parents value the role of early education beyond just school outcomes. However, other studies in the same context have emphasized how the perception of early education as too individualistic or inappropriate for learning affects parents’ hesitance to send their children to non-family-based early childcare (Yamamoto and Li, 2012). Parental involvement has seldom been studied in Chile (Lara and Saracostti, 2019) and even less in early education, beyond noting the importance of incorporating family into this process and acknowledging the culture and language of the home (Rolla and Rivadeneira, 2006).

When parents come from different cultural backgrounds than those of their children’s early educators, their beliefs and practices, educational levels, origins, expectations, and other family-related aspects can impact how children participate in early education (Fantuzzo et al., 2000; Kang et al., 2024; Xi and Wang, 2024; Yamamoto and Li, 2012). The more involved parents are, the better their children’s chances for academic success and social and emotional development (Arzubiaga et al., 2009; Barahona Cruz et al., 2023; Sylva et al., 2004). However, immigrant parents face unique challenges related to economic, cultural, social, and linguistic barriers (Barahona Cruz et al., 2023; Kalmijn, 2024; Kong et al., 2024; Pérez-Domínguez, 2024; Xi and Wang, 2024), which hinder their involvement in their children’s education. Several studies have shown that immigrant parents tend to exhibit low levels of school involvement across different stages of children’s education. This is often due to the fact that these parents typically have low educational levels, low incomes, and precarious work conditions, which negatively affect their motivation and ability to actively participate in their children’s schooling processes (Antony-Newman, 2018; Calzada et al., 2015; Friedman et al., 2006; González-Falcón et al., 2022).

The length of stay in the host society can also be a factor, as it influences knowledge and comprehension of the local culture, particularly the host country’s educational system (Mera-Lemp et al., 2025). Moreover, what is understood as parental involvement may be culturally divergent (Hoover-Dempsey et al., 2005; Keles et al., 2024) and, therefore, may not necessarily align with teachers’ expectations and perceptions of involvement. For instance, parents from collectivistic cultures tend to stress the value of respect and discipline; thus, they may be more likely to understand their role in complying with the school’s explicit requests. In contrast, individualistic cultures, characterized by a lesser distance to power in social relationships and a strong orientation toward goal achievement, may emphasize supporting children in fulfilling their personal aims and being more proactive in their relationships with teachers (Bornstein, 2017; Bornstein and Cote, 2006; Gallardo, 2019).

In Chile, according to the most recent data, it is estimated that 8.8% of the population is composed of foreigners, mainly from other Latin American and Caribbean countries (Encuesta de Caracterización Socioeconómica Nacional, 2022; Instituto Nacional de Estadísticas and Departamento de Extranjería y Migración, 2022; Servicio Jesuita a Migrantes, 2022). A total of 13% of this percentage comprises children and teenagers (Servicio Nacional de Migraciones, 2023), with currently 10,332 children enrolled in early education programs (Fondo de las Naciones Unidas para la Infancia and Instituto Nacional de Estadísticas, 2023); however, this number does not consider children born in Chile to immigrant parents or those with irregular immigration statuses, which means the magnitude of this phenomenon is invisibilized. Moreover, generally speaking, there is a 20% lower chance for immigrant children to attend early education centers compared to their Chilean peers (Ministerio de Relaciones Exteriores de Chile, 2024), despite the positive effect that early education has on social mobility and inclusion (Barnett and Frede, 2010; Cebolla Boado, 2008; Hughes, 2010; Kim and Kang, 2024; Turaşlı, 2016; Yamamoto and Li, 2012).

Literature has suggested that interculturally sensitive practices, which respond to the cultural and linguistic needs of families, may generate more effective communication between parents and teachers in early education (Barahona Cruz et al., 2023; Yamamoto and Li, 2012). Intercultural sensitivity is understood as the affective dimension of intercultural communication, encompassing the skills to manage the emotions that arise from intercultural exchanges and to express positive emotional responses (Chen and Starosta, 2000; Ruiz-Bernardo et al., 2024). It is essential for addressing the cultural differences of contemporary multicultural societies (Pastena et al., 2024), as it enables communication that recognizes and accepts these differences while also discovering cultural similarities (Martínez-Zelaya et al., 2020; Sell, 2017).

According to Chen and Starosta (2000), intercultural sensitivity encompasses different capabilities, such as individuals’ abilities to appropriately receive and respond to messages in encounters with others, adjusting their cognitive frames and behaviors during interactions with diverse individuals. Additionally, it requires a willingness to engage in intercultural communication. Another important aspect concerns individuals’ attitudes toward cultural differences, specifically in terms of respect and acceptance, as well as their confidence in achieving positive results in these intercultural exchanges. Positive emotional experiences through intercultural communication also play a key role in these processes, allowing individuals to construct positive expectations about future intercultural encounters, thereby motivating them to interact in multicultural settings. Conversely, the absence of these capabilities could lead to expectations of negative outcomes from intercultural interactions, affecting attitudes toward communicating with people from different cultural backgrounds and potentially leading to the avoidance of intergroup contact (Bhawuk et al., 2008; Ulrey and Amason, 2001).

Studies conducted with teachers have shown that their own development of intercultural sensitivity influences their attitudes toward integrating immigrant students and their families into the schools (Mera-Lemp et al., 2024). Additionally, it has been suggested that this capability is affected by cultural threats and negative emotions (Mera-Lemp et al., 2024), their own biases and attitudes (Keles et al., 2024), and cultural empathy (Ryu, 2022). Teachers serve as a bridge between families and early educational centers and must, therefore, adapt their pedagogical practices and communication to the individual and cultural characteristics and needs of both the children and the parents (Barahona Cruz et al., 2023; Katıtaş et al., 2024; Özdoğru et al., 2024).

On the other hand, immigrant parents actively carry their cultural beliefs and expectations and negotiate these with the norms of the host society (Yamamoto and Li, 2012). Their awareness of the importance of supporting their children’s development in early education can motivate them to adjust their beliefs and practices in school settings (Kong et al., 2024; Subramaniam, 2011). Yet, as posed earlier, teachers may not perceive parents as involved as they should be since they do not necessarily account for diverse approaches to what is considered involvement (Kalmijn, 2024; Keles et al., 2024; Massing et al., 2013; Sánchez-Suzuki Colegrove, 2022). Historically, Chilean public policies have viewed cultural differences as obstacles to overcome rather than strengths to consider (Castillo Lobos and Contreras Valeria, 2023); this translates into early education as well, where the curriculum pushes for multiculturalism and diverse approaches without providing the tools necessary to implement them.

In addition, intercultural sensitivity can be an asset in enhancing immigrant parents’ involvement in their children’s education. Daily contact with teachers and participation in school activities, such as parent-teacher meetings and extracurricular events, involve direct interaction with educators and school staff, as well as with local families (Hayakawa et al., 2013; Sohn and Wang, 2006). Even though there is scarce evidence about this relationship, recent studies have shown that cultural sensitivity plays a significant role in explaining immigrant parents’ school involvement, motivating them to interact with school members (Mera-Lemp et al., 2025). Additionally, studies conducted with immigrant school students (Lahoz i Ubach, and Cordeu Cuccia, 2021) have reported that high levels of intercultural sensitivity are associated with positive perceptions of the school climate. In the case of university students in multicultural educational contexts, it has been linked with high levels of involvement in both formal and informal academic activities (Tamam and Krauss, 2017).

However, other aspects could influence parents’ school participation. According to Hoover-Dempsey and Sandler’s (1997) model explaining parents’ involvement, explicit invitations to engage in the school community, a welcoming and warm school climate, and perceived self-efficacy and confidence are essential factors in promoting their commitment to children’s schooling processes (Ferrara, 2015; Murray et al., 2014; Subramaniam, 2011). Additionally, the perception of respectful treatment and positive attitudes toward their cultural identities and parenting styles from school staff could also enhance their relationships and participation at school (Antony-Newman, 2018; Hoover-Dempsey et al., 2005; Khalfaoui et al., 2020; Mera-Lemp et al., 2025).

On the contrary, immigrant parents often face several barriers in their relationships with schools. It has been argued that since the school’s role is related to transmitting the dominant group’s culture, these parents may perceive cultural discontinuities or clashes between the values, beliefs, and behavioral guidelines they want their children to learn and those taught by teachers (Antony-Newman, 2018; Bourdieu and Passeron, 1990; Epstein, 2018; Hornby and Lafaele, 2011). Likewise, the perception of adverse school climates that could expose children to various forms of exclusion appears to be a critical issue, especially for parents belonging to minority groups (Baker et al., 2016; Reynolds et al., 2015). Thus, some immigrant parents tend to prefer enrolling their children in schools with a high presence of foreign families, as this facilitates their integration (Eberhard and Lauer, 2019). Nevertheless, further research is needed to understand the barriers and facilitators influencing parental involvement and the role of parents’ intercultural sensitivity in early education.

The present study aimed to explore the variables that could predict immigrant parents’ level of parental involvement in their children’s school activities in Chilean early education centers. It is expected that parents’ involvement is explained by their levels of intercultural sensitivity (H1) and also that this variable has more explicative potential than parents’ educational level (H2.1) and their time of residence in Chile (H2.2).

## 2 Materials and methods

### 2.1 Participants

The study used a non-probabilistic convenience sampling method, considering that the participants were immigrants with children in their care attending early education centers at the pre-kindergarten and kindergarten levels in Chile. The sample was composed of 347 immigrant parents; their sociodemographic characteristics are explained in the following table, distributed by their level of parental involvement. The parents were recruited by professional interviewers who visited areas near educational institutions (*k* = 90 schools) in the city of Santiago. Additionally, the study participants could recommend that others in their communities join the research. Each participant was interviewed individually, given a questionnaire, and assisted in case of doubts. The main inclusion criteria were as follows: (a) being of immigrant origin, (b) being the parent of a student enrolled in pre-kindergarten or kindergarten at an educational institution in Santiago, and (c) speaking Spanish fluently. The present study has received ethical permission from the Research Ethics Committee of Universidad Alberto Hurtado, considering all the standards of the Declaration of Helsinki.

### 2.2 Instruments

Descriptive statistics: each participant answered a series of questions that included their gender, age, educational level, country of origin, and the educational level of their son or daughter. Table 1 presents a complete description of the sample characteristics. For the multinomial logistic regression, the category “incomplete high school” collapsed with “complete high school,” termed “high school” due to the low frequency of the former. Additionally, participants from nationalities not mentioned in Table 1 were excluded for the same reason. Finally, time in Chile was transformed into quartiles (min = 0 and max = 16 years in Chile), with 2, 5, and 7 years of residence representing the 25th, 50th, and 75th percentiles, respectively.

**Table 1:** Sociodemographic characteristics by levels of parental involvement.

Variables	Almost never (*N* = 74)	Frequently (*N* = 116)	Always (*N* = 157)	Total (*N* = 347)
Gender Masculine	18 (24.3%)	27 (23.3%)	41 (26.1%)	86 (24.8%)
Feminine	56 (75.7%)	89 (76.7%)	116 (73.9%)	261 (75.2%)
Educative level incomplete high school	8 (10.8%)	9 (7.8%)	7 (4.5%)	24 (6.9%)
Complete high school	30 (40.5%)	49 (42.2%)	92 (58.6%)	171 (49.3%)
Technical/Professional education	29 (39.2%)	47 (40.5%)	25 (15.9%)	101 (29.1%)
University education	7 (9.5%)	11 (9.5%)	33 (21%)	51 (14.7%)
Country of origin Venezuela	31 (41.9%)	54 (46.6%)	81 (51.6%)	166 (47.8%)
Peru	15 (20.3%)	21 (18.1%)	33 (21%)	69 (19.9%)
Colombia	7 (9.5%)	21 (18.1%)	17 (10.8%)	45 (13%)
Haiti	18 (24.3%)	11 (9.5%)	13 (8.3%)	42 (12.1%)
Other	3 (4.1%)	9 (7.8%)	13 (8.3%)	25 (7.3%)
Age	33.24 (6.15)	33.48 (6.24)	33.72 (6.62)	33.53 (6.38)
Time of residence (years)	4.40 (2.66)	4.83 (2.99)	5.55 (3.02)	5.04 (2.96)

Numbers with the % symbol represent the percentage of cases (i.e., frequency) within the total of each variable. The numbers with decimals and those without the % symbol, represent the mean and the standard deviation, respectively.

Parental Involvement Scale (Valdés Cuervo et al., 2009): this instrument assesses parental involvement in their children’s school activities and the educational process related to communication with the center (e.g., *attending parent and/or family meetings*) and participation (e.g., *participating in raffles, celebrations, and activities organized by the educational establishment*), as well as communication with educators (e.g., *talking to the teacher about your child’s learning*) and the child (e.g., *talking to your child about their classmates*). We used 17 of the 23 original items, given that the instrument also included items about the context of the school that were not pertinent to early education. The scale has a Likert format ranging from 1 (Never) to 4 (Always), with adequate reliability (*α* = 0.90; *ω* = 0.92, *X*_(113)_ = 243.830, *p* < 0.001; CFI 0.991; TLI 0.989; RMSEA 0.060 [0.050; 0.070]). Given that it is a discrete scale and few participants indicated “never” being involved, we summed the answers of the items and, based on the distribution, created three categories: (a) 1 (*almost never*): total score from 17 to 34; (b) 2 (*frequently*): total score from 35 to 67; (c) 3 (*always*): total score of 68. These three categories were used for the analysis.

*The Intercultural Sensitivity Scale* (Chen and Starosta, 2000), along with the Spanish version validated by Martínez-Zelaya et al. (2024), assesses the affective dimension of intercultural communication in terms of how effectively and respectfully individuals interact with people from diverse cultural backgrounds (e.g., *“When I speak with people from different cultures, I try to gather as much information as possible”*; e.g., *“When I talk to a person from another culture, I enjoy the differences between us.”*). It consists of 24 Likert-type items with four answer options (1: Totally disagree, to 5: Totally agree), demonstrating good reliability (*α* = 0.81; *X*_(166)_ = 252.106, *p* < 0.001; CFI 0.965; TLI 0.942; RMSEA 0.040 [0.03; 0.05]).

Facilitators and barriers (ad hoc): finally, we constructed proxies for the facilitators and barriers that immigrant parents may encounter when participating in their child’s educational process. These were based on the literature and interviews conducted with immigrant parents during the validation of the instruments. There were seven facilitators of parental participation in school activities: a *welcoming attitude from educators, respectful treatment by educators, the child feeling integrated with the rest of the class, educators attempting to understand their culture, customs, and parenting styles, a positive climate among parents, the school having other students from immigrant families, and the school offering a curriculum that integrates cultural diversity*. Additionally, six barriers were *identified: the child feeling rejected by peers, parents feeling rejected by other parents, feeling questioned by teachers for being different from Chileans, the school lacking a special approach for immigrant families, a lack of understanding of the Chilean educational system, and a short duration of stay in Chile*. The question posed was, “*Considering your experience, what do you think are the factors that facilitate/hinder the participation of immigrant parents in the education of their children*?” Each of these variables was rated with a value of 0 (i.e., does not affect) or 1 (i.e., does affect).

### 2.3 Analysis

First, we performed descriptive analyses on the levels of participation in school activities, categorized into three levels (i.e., almost never [1], frequently [2], and always [3]), according to the characteristics of the participants (see Table 1). Additionally, we complemented these descriptions with either chi-squared or ANOVA tests—for categorical or numerical variables, respectively— to determine whether participation varied based on the characteristics of the sample. Subsequently, to test the main hypotheses, we conducted multinomial logistic regressions to predict the probability of parental involvement at different levels (i.e., almost never, frequently, and always). To achieve this, we used the descriptive variables measured as predictors, the parents’ levels of intercultural sensitivity, and, finally, the facilitators and barriers to participation indicated by the participants. To avoid multicollinearity and saturated models, we conducted these predictions in two separate models: one including facilitators and the other including barriers. All analyses were performed using SPSS v27 software.

## 3 Results

### 3.1 Descriptive analyses

Table 1 shows the levels of parental involvement in their children’s education among the participants, segmented by gender, educational level, country of origin, age, and time of residence in Chile. It is evident that women exhibited higher levels of involvement (75.2%), with the majority of parents having completed high school (49.3%). Venezuelans constituted the largest group across all levels of involvement (47.8%), followed by Peruvians (19.9%) and Colombians (13%). The average age of the parents remains consistent across categories, at approximately 33 years, with a mean residence time in Chile of approximately 5.04 years.

Chi-squared and ANOVA tests revealed that neither gender [*χ*^2^(2) = 0.299, *p* = 0.861] nor age [*F*(2, 344) = 0.035, *p* < 0.966; Eta-squared = 0.000] was related to parental involvement among the parents in the sample. Conversely, parental educational level [*χ*^2^(4) = 29.66, *p* < 0.001], in favor of the university level, and Venezuelan nationality [*χ*^2^(6) = 16.25, *p* = 0.012] were associated with higher levels of parental involvement. The time of residence in Chile was not significantly associated with parental involvement; however, there was a tendency in the results [*F*(2, 344) = 2.888, *p* = 0.057; Eta-squared = 0.017].

### 3.2 Main analyses

We conducted two multinomial logistic regression analyses to predict the parental involvement of immigrant families in early education based on the level of intercultural sensitivity (i.e., the common predictor) and the possible factors that can be facilitators (i.e., Table 2) and barriers (i.e., Table 3) to parents’ participation. Parental involvement consisted of three categories of participation in their child’s educational process: almost never, frequently, and always (the reference category). Two models were executed, one with sociodemographic variables, intercultural sensitivity, and facilitators as predictors and another one with barriers as predictors. As can be seen in both models and for both contrasts, the parents’ levels of intercultural sensitivity were significant predictors of parental involvement.

**Table 2:** Involvement of immigrant families in the educational process of their children (facilitator model).

Predictor	*B*	*SE*	*p*	OR [95% CI]
Contrast 2: Almost never vs. Always				
Age	0.041	0.029	0.160	1.042 [0.984, 1.103]
Gender^1^	−0.070	0.393	0.859	0.933 [0.432, 2.014]
Country of origen^2^				
Venezuela	−0.780	0.681	0.253	0.459 [0.121, 1.744]
Peru	−0.434	0.648	0.503	0.648 [0.182, 2.306]
Colombia	−0.673	0.789	0.394	0.510 [0.109, 2.395]
Educational level^3^				
High school	0.341	0.593	0.565	1.407 [0.440, 4.501]
Technical/professional	1.642	0.594	0.006	5.164 [1.611, 16.547]
Time in Chile^4^				
0–2 years	0.843	0.621	0.175	2.323 [0.688, 7.841]
2–5 years	0.068	0.600	0.910	1.070 [0.330, 3.469]
5–7 years	0.626	0.614	0.308	1.870 [0.561, 6.233]
School level (child)^5^	−0.970	0.376	0.010	0.379 [0.182, 0.791]
Intercultural Sensitivity	−1.386	0.424	0.001	0.250 [0.109, 0.575]
Facilitator 1	0.426	0.571	0.456	1.531 [0.499, 4.691]
Facilitator 2	0.443	0.461	0.336	1.558 [0.631, 3.845]
Facilitator 3	0.237	0.472	0.615	1.268 [0.502, 3.200]
Facilitator 4	−0.453	0.415	0.275	0.636 [0.282, 1.435]
Facilitator 5	−0.580	0.445	0.193	0.560 [0.234, 1.340]
Facilitator 6	1.292	0.422	0.002	3.641 [1.594, 8.317]
Facilitator 7	0.412	0.455	0.365	1.509 [0.619, 3.679]
Contrast 2: Frequently vs. Always				
Age	−0.002	0.025	0.940	0.998 [0.949, 1.049]
Gender^1^	−0.218	0.341	0.524	0.804 [0.412, 1.571]
Country of origen^2^				
Venezuela	0.321	0.630	0.610	1.379 [0.401, 4.741]
Peru	0.169	0.622	0.785	1.185 [0.350, 4.007]
Colombia	0.868	0.676	0.199	2.382 [0.634, 8.958]
Educational level^3^				
High school	0.216	0.465	0.643	1.241 [0.499, 3.086]
Technical/professional	1.480	0.477	0.002	4.392 [1.723, 11.195]
Time in Chile^4^				
0–2 years	−0.268	0.507	0.597	0.765 [0.283, 2.067]
2–5 years	−0.168	0.463	0.717	0.845 [0.341, 2.094]
5–7 years	−0.002	0.476	0.996	0.998 [0.392, 2.538]
School level (child)^5^	−0.471	0.307	0.125	0.624 [0.342, 1.139]
Intercultural Sensitivity	−0.766	0.347	0.027	0.465 [0.235, 0.918]
Facilitator 1	0.448	0.501	0.371	1.566 [0.587, 4.179]
Facilitator 2	−0.056	0.419	0.894	0.946 [0.416, 2.148]
Facilitator 3	0.265	0.416	0.524	1.303 [0.577, 2.944]
Facilitator 4	0.241	0.369	0.513	1.273 [0.618, 2.622]
Facilitator 5	−0.996	0.392	0.011	0.369 [0.171, 0.796]
Facilitator 6	1.279	0.362	0.000	3.594 [1.769, 7.304]
Facilitator 7	0.012	0.397	0.976	1.012 [0.465, 2.202]

B, SE, and OR [95% CI] indicate the regression coefficients, standard errors and the odds ration with their 95% confidence intervals, respectively. ^1^Gender is a categorical variable being 0 male and 1 female. ^2^Country of origin is a dummy comparison with a reference of being from Haiti. ^3^Educational level is a dummy comparison with a reference of having the highest level, that is university level. ^4^Time in Chile was transformed to quartiles (min = 0 and max = 16 years in Chile) being 2, 5 and 7 years of residence the percentiles 25, 50 and 75, respectively. 5, Pre-Kindergarten vs. Kindergarten. The facilitators were 1: A welcoming attitude on the part of the educators; 2: Respectful treatment by the educators; 3: That your child feels integrated with the rest of the class; 4: That the educators try to understand their culture, customs, parenting styles; 5: A good climate among parents; 6: That the school has other students from immigrant families; 7: That the school has a curriculum that integrates cultural diversity.

**Table 3:** Involvement of immigrant families in the educational process of their children (barrier model).

Predictor	*B*	*SE*	*p*	OR [95% CI]
Contrast 1: Almost never vs. Always				
Age	0.042	0.029	0.152	1.043 [0.985, 1.104]
Gender^1^	−0.041	0.390	0.916	0.960 [0.447, 2.059]
Country of origen^2^				
Venezuela	−1.047	0.661	0.113	0.351 [0.096, 1.281]
Peru	−0.716	0.640	0.264	0.489 [0.139, 1.715]
Colombia	−0.761	0.775	0.326	0.467 [0.102, 2.133]
Educational level^3^				
High school	0.537	0.591	0.364	1.710 [0.537, 5.447]
Technical/professional	1.621	0.594	0.006	5.058 [1.580, 16.194]
Time in Chile^4^				
0–2 years	0.983	0.621	0.113	2.673 [0.792, 9.022]
2–5 years	0.189	0.586	0.747	1.208 [0.383, 3.805]
5–7 years	0.611	0.601	0.309	1.843 [0.567, 5.990]
School level (child)^5^	−0.919	0.371	0.013	0.399 [0.193, 0.826]
Intercultural Sensitivity	−1.203	0.425	0.005	0.300 [0.131, 0.691]
Barrier 1	−0.573	0.621	0.356	0.564 [0.167, 1.904]
Barrier 2	0.192	0.421	0.649	1.211 [0.531, 2.764]
Barrier 3	−0.124	0.462	0.788	0.883 [0.357, 2.185]
Barrier 4	1.334	0.427	0.002	3.796 [1.642, 8.775]
Barrier 5	−0.622	0.442	0.160	0.537 [0.226, 1.278]
Barrier 6	0.634	0.417	0.128	1.886 [0.833, 4.270]
Contrast 2: Frequently vs. Always				
Age	0.005	0.024	0.841	1.005 [0.958, 1.054]
Gender^1^	−0.125	0.330	0.705	0.883 [0.462, 1.685]
Country of origen^2^				
Venezuela	0.208	0.623	0.738	1.232 [0.364, 4.174]
Peru	−0.051	0.614	0.934	0.951 [0.285, 3.167]
Colombia	0.723	0.676	0.285	2.060 [0.548, 7.747]
Educational level^3^				
High school	0.435	0.463	0.347	1.545 [0.624, 3.825]
Technical/professional	1.559	0.474	0.001	4.756 [1.877, 12.053]
Time in Chile^4^				
0–2 years	−0.064	0.497	0.897	0.938 [0.354, 2.483]
2–5 years	−0.017	0.445	0.970	0.983 [0.411, 2.351]
5–7 years	−0.035	0.469	0.941	0.966 [0.385, 2.424]
School level (child)^5^	−0.459	0.303	0.130	0.632 [0.349, 1.144]
Intercultural Sensitivity	−0.679	0.339	0.045	0.507 [0.261, 0.985]
Barrier 1	−0.334	0.516	0.517	0.716 [0.260, 1.969]
Barrier 2	0.215	0.365	0.556	1.239 [0.606, 2.533]
Barrier 3	0.198	0.389	0.611	1.219 [0.569, 2.611]
Barrier 4	0.382	0.355	0.282	1.465 [0.731, 2.939]
Barrier 5	−0.709	0.382	0.063	0.492 [0.233, 1.040]
Barrier 6	0.911	0.354	0.010	2.487 [1.243, 4.978]

B, SE, and OR [95% CI] indicate the regression coefficients, standard errors and the odds ration with their 95% confidence intervals, respectively. ^1^Gender is a categorical variable being 0 male and 1 female. ^2^Country of origin is a dummy comparison with a reference of being from Haiti. ^3^Educational level is a dummy comparison with a reference of having the highest level, that is university level. ^4^Time in Chile was transformed to quartiles (min = 0 and max = 16 years in Chile) being 2, 5, and 7 years of residence the percentiles 25, 50, and 75, respectively. 5, Pre-Kindergarten vs. Kindergarten. The barriers were 1: That their child feels rejected by peers; 2: That they feel rejected by other parents; 3: That they feel questioned by the teachers for being different from Chileans; 4: The school does not have a special approach for immigrant families; 5: They do not understand the Chilean educational system; 6: That they have only been in Chile for a short time.

In the first model (Table 2), the predictors examined whether intercultural sensitivity explained parental involvement in the presence of other predictors, such as structural factors (age, gender, country of origin, educational level, time in Chile, and the child’s early education level) and facilitators of parental participation in school activities (*a welcoming attitude on the part of the educators; respectful treatment by the educators; ensuring that the child feels integrated with the rest of the class; the educators’ efforts to understand families’ cultures, customs, and parenting styles; a good climate among parents; having other students from immigrant families at the school; and a curriculum that integrates cultural diversity*). This model with these variables accounted for more than 30% of the variability in parental involvement (*R*^2^_Nagelkerke_ = 0.322).

For the contrast between “*almost never*” and “*always,*” it was shown that an increase in intercultural sensitivity was associated with 75% lower odds of parents *almost never* being involved in their children’s educational process compared to *always* being involved (OR = 0.250, 95% CI [0.109, 0.575], *p* = 0.001). For the facilitators, only one predictor was significant: the presence of other immigrant families in the center. Parents who selected this facilitator had 3.64 times higher odds of *almost never* being involved compared to being *always* involved (OR = 3.641, 95% CI [1.594, 8.317], *p* = 0.002). Additionally, having a child at a higher educational level (kindergarten) was linked to 62.1% lower odds of being *almost never* involved (OR = 0.379, 95% CI [0.182, 0.791], *p* = 0.010). Finally, parents with technical or professional education (vs. university level) had 5.16 times higher odds of *almost never* being involved in their children’s educational process (OR = 5.164, 95% CI [1.611, 16.547], *p* = 0.006).

For the contrast between “*frequently*” and “*always*,” intercultural sensitivity was again a significant predictor (OR = 0.465, 95% CI [0.235, 0.918], *p* = 0.027), which was associated with 53.5% lower odds of reporting *frequent* involvement instead of *always*. Additionally, the facilitator of a good climate among parents was associated with 63.1% lower odds of being *frequently* involved compared to *always* (OR = 0.369, 95% CI [0.171, 0.796], *p* = 0.011). As in the other contrast, the choice of facilitator related to the presence of other immigrant families in the school was associated with 3.59 times higher odds of being *frequently* involved (OR = 3.594, 95% CI [1.769, 7.304], *p* < 0.001). Also, as in the previous contrast, parents with technical or professional education showed lower levels of participation, with 4.39 times higher odds of reporting being *frequently* involved instead of *always* (OR = 4.392, 95% CI [1.723, 11.195], *p* = 0.002).

In summary, for this model, parents with higher levels of intercultural sensitivity are more likely to always participate, thereby reducing the odds of participating almost never or only frequently. Other significant predictors include having a child at higher levels of early education. As facilitators, parents who indicated having a good climate among parents were more likely to be always involved than to participate only frequently. Conversely, parents who chose to facilitate with more immigrant families at the center were associated with lower levels of participation, as were those with technical or professional education. This last variable emerged as the most important predictor of parental involvement in their children’s educational processes. No significant associations were found for age, gender, or time residing in Chile in either contrast or for other facilitators.

In the second model (Table 3), the same variables were included; however, instead of facilitators, we introduced barriers to parental involvement as predictors: (*that their child feels rejected by peers; that they feel rejected by other parents; that they feel questioned by the teachers for being different from Chileans; that the school does not have a special approach for immigrant families; that they do not understand the Chilean educational system; that they have only been in Chile for a short time*). The variables introduced to the model accounted for more than 30% of the variability of parental involvement (*R*^2^_Nagelkerke_ = 0.311).

In contrast to “almost never” and “always,” parents’ levels of intercultural sensitivity also explain parental involvement in the children’s educational process. Specifically, parents with lower intercultural sensitivity were significantly more likely to report lower levels of involvement, whereas parents with higher intercultural sensitivity were 70% more likely to report being *always* involved rather than *almost never* (OR = 0.300, 95% CI [0.131, 0.691], *p* = 0.005). Regarding barriers, parents who believed that the school lacked a special approach for immigrant families were 3.79 times more likely to report low involvement (OR = 3.796, 95% CI [1.642, 8.775], *p* = 0.002). As in the model for facilitators, parents with a technical or professional education had significantly higher odds of reporting *almost never* being involved in educational activities (OR = 5.058, 95% CI [1.580, 16.194], *p* = 0.006). Similarly, parents with children in higher school levels, such as kindergarten, had lower odds of reporting *almost never* being involved compared to parents with children in pre-kindergarten (OR = 0.399, 95% CI [0.193, 0.826], *p* = 0.013).

For the contrast between “frequently” and “always” regarding parental involvement, intercultural sensitivity again emerges as a significant predictor. Higher intercultural sensitivity is associated with 1.97 times lower odds of being *frequently* involved compared to being *always involved* (OR = 0.507, 95% CI [0.261, 0.985], *p* = 0.045). In all circumstances of parental involvement, higher intercultural sensitivity in parents predicts greater odds of always participating in their children’s educational process. In this contrast, the barrier of being in Chile for a shorter time proves significant, with 2.48 times higher odds of reporting *frequent* involvement compared to being *always involved* (OR = 2.487, 95% CI [1.243, 4.978], *p* = 0.010). As in the previous contrast, higher educational levels of parents are significantly associated with greater odds of reporting only *frequent* involvement (OR = 4.756, 95% CI [1.877, 12.053], *p* = 0.001).

In summary, for the model with the barriers, parents with higher levels of intercultural sensitivity showed higher levels of involvement in their children’s educational processes in both contrasts. Similar to the other model, parents with children at a higher educational level also have greater odds of consistently participating. On the other hand, viewing the center’s lack of a special approach for immigrant families as a barrier discourages parental participation, nearly to the extent of never versus always. Furthermore, parents who identify being in Chile for a short time as a barrier tend to participate more frequently than those who participate consistently. Additionally, the most significant predictor appears to be the technical/professional educational level; these parents tend to participate less often than those with a university education.

Predictors such as age, gender, the time residing in Chile, and other barriers did not show significant associations in either contrast.

## 4 Discussion

This study aimed to explore the relationship between intercultural sensitivity and immigrant parents’ involvement in their children’s early education. Our results emphasize the significant role of parents’ intercultural communication skills in enhancing their active participation in children’s education, encompassing both school and home tasks. Additionally, the lack of specific approaches for immigrant families, combined with their shorter length of stay in Chile and the high presence of immigrant families in schools, poses significant barriers to the participants’ involvement. Moreover, the perception of a positive climate among parents appears to facilitate their participation.

Our central hypothesis was that parental involvement would be explained by their levels of intercultural sensitivity (H1). Furthermore, this variable would have greater explanatory potential than the educational level of the parents (H2.1) and their length of residence in Chile (H2.2). Relevant sociodemographic variables, including gender, age, educational level, nationality, length of residence in Chile, and the grade in which their children were enrolled, were considered. Additionally, factors that could facilitate or hinder parental participation were evaluated in separate models.

We performed two multinomial logistic regression models for parental involvement, contrasting being *almost never* (1) or *frequently* (2) involved against *always* (3). Both models considered the common variables of intercultural sensitivity and sociodemographic factors. The first model included facilitators as predicting variables, while the second model included barriers. Both models confirmed the first hypothesis, showing that intercultural sensitivity was a significant predictor of higher levels of parental involvement in their children’s educational processes.

In the first model, which included facilitators, more than 30% of the variability in parental involvement was explained. For both contrasts [1 vs. 3; 2 vs. 3], parents’ levels of intercultural sensitivity were significant predictors of parental involvement. Specifically, higher levels of intercultural sensitivity increase the likelihood of greater involvement in children’s educational processes. This supports other studies that showed that high participation in the education of immigrant parents is related to the intercultural sensitivity of different members of the educational community (Cala et al., 2018; Massing et al., 2013; Mera-Lemp et al., 2024, 2025).

Particularly, these results suggest that participants’ ability to recognize cultural differences, maintain a respectful attitude toward them, and adjust their own cultural frames and behaviors through intercultural interactions leads to enjoyment in these encounters and confidence in communicating in intercultural settings. This appears to motivate parents to engage in school activities and participate in their children’s educational processes. Therefore, immigrant parents can also benefit from developing their communication skills in relationships with other cultures, particularly with the Chilean culture, and fostering their cultural sensitivity through interactions at early educational establishments.

Additionally, compared to parents with the highest level of education (i.e., university level), those with technical or professional levels were five times more likely to be uninvolved in their children’s education. Studies on minority group parents’ school involvement have systematically shown that their educational backgrounds play a key role in their participation in their children’s schooling (Calzada et al., 2015; Friedman et al., 2006; González-Falcón et al., 2022). Parents with high educational levels tend to perceive themselves as more confident and qualified to be involved in their children’s education. Similarly, their cultural capital tends to align more coherently with the content that schools transmit through education (Antony-Newman, 2018; Calzada et al., 2015; Bourdieu and Passeron, 1990; Epstein, 2018; Hornby and Lafaele, 2011).

Finally, regarding the facilitators in the model, the presence of other immigrant families in the school was associated with lower parental involvement. While this finding requires further research, one possible explanation is that centers with a high presence of immigrant families may face greater challenges in managing cultural diversity (Calzada et al., 2015; Glock et al., 2019; Gutentag et al., 2018), which could contribute to parents’ disengagement from participation (Kalmijn, 2024). This aligns with studies showing that immigrant families often face challenges in balancing the expectations that early education in the host society places on them with their own cultural values and traditions (Yamamoto and Li, 2012; Kong et al., 2024; Subramaniam, 2011). Another possible explanation is that in the Chilean context, studies conducted with Latin American immigrants have shown a high interest in integrating into the local society while maintaining their cultural identities but, at the same time, adopting some aspects of the host culture (Mera-Lemp et al., 2021; Sirlopú and Renger, 2020). Thus, participating in school communities composed mainly of other immigrants could be less attractive and discourage them from participating.

Relatedly, another significant facilitator was maintaining a positive climate among parents, which increased the probability of frequent to constant participation by 39%. Parents’ perceptions of the school climate could lead them to commit extensively to their children’s education by providing positive emotional experiences and opportunities for building social relationships (Murray et al., 2020). Together, these facilitators underscore the importance of immigrant parents integrating as equals with all parents in the class to enhance their likelihood of consistently being involved in their children’s educational processes (Calzada et al., 2015; Baker et al., 2016; Khalfaoui et al., 2020; Reynolds et al., 2015; Sohn and Wang, 2006).

The analysis that introduced the barriers instead of facilitators showed similar results. Again, parents’ levels of intercultural sensitivity were significant predictors of parental involvement in the educational processes in early education. In detail, greater levels of this variable increased parental involvement by 70% from *never* to *always* and approximately 50% from *frequently* to *always*. Considering the reported barriers to participation, a barrier that appeared significant when there was already parental involvement (i.e., the contrast of frequently vs. always) was that being in Chile for a shorter time decreased the parents’ involvement. As a hypothesis, this could be due to the fact that parents who have lived in the country for a shorter time may have a more limited understanding of the educational system and the host culture, which could discourage them from engaging in educational processes (Antony-Newman, 2018). It is worth noting, however, that among the sociodemographic variables assessed, the length of stay in the country was not significant in the models. Therefore, it is possible that this aspect is perceived as a barrier but is not actually one for those who are involved.

Another significant barrier to predicting parental involvement was that the educational institution did not have a specific approach for immigrant families, which reduced parental involvement by 3.7 times. Clearly, this issue is related to the presence of other students from immigrant families and a positive climate among parents. Altogether, this calls for an early education context that intentionally welcomes immigrant families. This is also relevant for future learning outcomes, as the meta-analysis by Ma et al. (2016) demonstrated. The relationship between parental involvement in early education and learning outcomes was strong (0.509) but considerably weaker among minority children. The study suggests that barriers such as cultural differences, language, and socioeconomic factors might diminish the impact of parental involvement. All of this can be addressed by developing intercultural sensitivity within the educational community, as this can effectively enhance parents’ participation, while structural variables are difficult to change.

Although intercultural sensitivity has always been important, in both comparisons, parents’ educational level had the strongest influence. In the case of frequent versus always, intercultural sensitivity was the weakest variable predicting the levels of parental involvement.

This study has several limitations that should be considered. First, it employed a non-random, cross-sectional design, which limits the ability to capture the dynamic nature of parental involvement over time. Parents’ participation in their children’s educational processes evolves as they gain familiarity with the teachers, other parents, the school environment, and the Chilean educational system. Thus, future studies could use a longitudinal approach to provide deeper insights into these changes. Second, the study excluded immigrant parents who do not speak Spanish (e.g., Haitian parents), a group likely to experience greater cultural differences and face one of the most significant barriers to school participation: language comprehension. Future research should address these gaps to gain a more comprehensive understanding of how the barriers associated with this limitation relate to parental involvement in their children’s early education educational processes. A third limitation of this study is that intercultural sensitivity was analyzed using only the total score without examining its different dimensions. Exploring these dimensions separately could provide a better understanding of how specific aspects of intercultural sensitivity—such as interaction engagement, respect for cultural differences, or interaction confidence—influence immigrant parents’ participation in their children’s educational processes.

Despite these limitations, our findings suggest that intercultural sensitivity can be important in increasing parental involvement in early education. Our results emphasize the importance of educational contexts that acknowledge the differences within immigrant families and their willingness to engage in respectful and interested communication, thereby promoting positive school climates. Furthermore, given that this study suggests that the management of cultural diversity by the centers could be an important asset in encouraging parents to actively participate, it will be interesting to explore the possible role of cultural diversity in the school climate on their levels of involvement.

Finally, our results suggest that educational policies should consider the development of intercultural sensitivity as an important asset for immigrant parents’ involvement. This could be achieved by including training in this capability in early education teachers’ curricula. Also, the creation of interventions aimed at increasing parents’ intercultural competencies could have an important impact, including on those who belong to the receiving country. This can improve intergroup relationships in order to promote the value of diversity within educational communities, helping overcome the actual difficulties.

Even though there is a lack of evidence regarding intervention programs to improve these capacities in immigrant parents, it is important to consider the role of the construction and implementation of reception protocols for immigrant families. These protocols could include information about the center’s functioning, the norms, and the expectations for parents’ roles. Similarly, they should consider cultural cues to understand interactions among parents and educators and guide them on how to obtain support from the school staff, including explicit invitations to participate and propose ideas to better understand their own cultures. However, further research is needed to design and develop these types of interventions, including, for example, qualitative studies to deeply understand immigrant parents’ participation in early education.

## Data Availability

The raw data supporting the conclusions of this article will be made available by the authors, without undue reservation.

## References

[ref2] Antony-Newman M. (2018). Parental involvement of immigrant parents: a meta-synthesis. Educ. Rev. 71, 362–381. doi: 10.1080/00131911.2017.1423278, PMID: 40101104

[ref3] Arzubiaga A. E. Noguerón S. C. Sullivan A. L. (2009). The education of children in Im/migrant families. Rev. Res. Educ. 33, 246–271. doi: 10.3102/0091732X08328243

[ref4] Baker T. L. Wise J. Kelley G. Skiba R. J. (2016). Identifying barriers: creating solutions to improve family engagement. Sch. Community J. 26, 161–184.

[ref5] Barahona Cruz Y. M. Sánchez Méndez J. J. De Ramírez Andrade M. L. Verdesoto Suárez L. F. (2023). Importancia de la familia en el aprendizaje preescolar. Polo del Conocimiento: Revista científico - profesional 8, 2835–2848. Available at: https://polodelconocimiento.com/ojs/index.php/es/article/view/5447 (Accessed January 28, 2025).

[ref6] Barnett W. S. Frede E. C. (2010). The promise of preschool why we need. Am. Educ. 40, 21–29.

[ref7] Bhawuk D. Sakuda K. H. Munusamy V. P. Intercultural competence development and triple-loop cultural learning: toward a theory of intercultural sensitivity. In Ang S. Dyne L. Van (Eds.), Handbook of cultural intelligence: theory, measurements and implications. Armonk, NY: M.E. Sharpe (2008) 342–355

[ref8] Bornstein M. H. (2017). Parenting in acculturation: two contemporary research designs and what they tell us. Curr. Opin. Psychol. 15, 195–200. doi: 10.1016/j.copsyc.2017.03.020, PMID: 28813262 PMC5604855

[ref9] Bornstein M. H. Cote L. R. (2006). “Parenting cognitions and practices in the acculturative process” in Acculturation and parent-child relationships: Measurement and development. eds. Bornstein M. H. Cote L. R. (Lawrence Erlbaum Associates Publishers), 173–196.

[ref10] Bourdieu P. Passeron J. C. (1990). Reproduction in education, society and culture, transl. R Nice. Newbury. Park, CA: Sage.

[ref11] Cala V. C. Soriano-Ayala E. López-Martínez M. J. (2018). Attitudes toward refugees and inclusive European citizenship. Analysis for an intercultural educational proposal with teachers in training. Relieve 24, 1–13. doi: 10.7203/relieve.242.13320

[ref12] Calzada E. J. Huang K. Y. Hernandez M. Soriano E. Acra C. F. Dawson-McClure S.. (2015). Family and teacher characteristics as predictors of parent involvement in education during early childhood among afro-Caribbean and Latino immigrant families. Urban Educ. 50, 870–896. doi: 10.1177/0042085914534862, PMID: 26417116 PMC4582786

[ref14] Castillo Lobos L. Contreras Valeria C. (2023). ¡Que no le falte la fruta al niño! Las prácticas de alimentación como acción política de las madres migrantes haitianas en Chile. Rev. Pueblos Front Digital 18, 1–30. doi: 10.22201/cimsur.18704115e.2023.v18.667

[ref15] Cebolla Boado H. (2008). Del preescolar a las puertas de la universidad. Un análisis de las trayectorias escolares de los estudiantes inmigrantes en Francia. Rev. Int. Sociol. 66, 79–103. doi: 10.3989/ris.2008.i51.110

[ref16] Chen G.-M. Starosta W. J. (2000). The development and validation of the intercultural sensitivity scale. Hum. Commun. 3, 3–14.

[ref18] Eberhard J. P. Lauer C. M. (2019). Diferencias en la elección de establecimiento educacional para la población local e inmigrantes: Caso chileno. Estudios Pedagógicos 45, 29–45. doi: 10.4067/S0718-07052019000200029

[ref19] Encuesta de Caracterización Socioeconómica Nacional. (2022). Available online at: https://observatorio.ministeriodesarrollosocial.gob.cl/encuesta-casen-2022 (Accessed January 28, 2025).

[ref20] Epstein J. L. (2018). School, family, and community partnerships: Preparing educators and improving schools. New York: Routledge.

[ref21] Fantuzzo J. Tighe E. Childs S. (2000). Family involvement questionnaire: a multivariate assessment of family participation in early childhood education. J. Educ. Psychol. 92, 367–376. doi: 10.1037/0022-0663.92.2.367

[ref22] Ferrara M. M. (2015). Parent involvement facilitators: unlocking social capital wealth. Sch. Community J. 25, 29–51.

[ref23] Fondo de las Naciones Unidas para la Infancia and Instituto Nacional de Estadísticas. (2023). Available online at: https://www.unicef.org/chile/media/8721/file/Informe%20migrantes%20INE%20V15.pdf (Accessed January 28, 2025).

[ref9001] Friedman B. A. Bobrowski P. E. Geraci J. (2006). Parents’ school satisfaction: Ethnic similarities and differences. J. Edu. Admin. 44, 471–486. doi: 10.1108/09578230610683769, PMID: 30238968

[ref24] Gallardo A. M. (2019). Cogniciones y prácticas parentales en contexto de migración. Summa Psicol. 16, 121–129. doi: 10.18774/0719-448x.2019.16.412

[ref25] Glock S. Kovacs C. Pit-ten Cate I. (2019). Teachers’ attitudes towards ethnic minority students: effects of schools’ cultural diversity. Br. J. Educ. Psychol. 89, 616–634. doi: 10.1111/bjep.12248, PMID: 30238968

[ref27] González-Falcón I. Arroyo-González M. J. Berzosa-Ramos I. Dusi P. (2022). I do the best I can: the role of immigrant parents in their children’s educational inclusion. Front. Educ. 7:1006026. doi: 10.3389/feduc.2022.1006026

[ref29] Gutentag T. Horenczyk G. Tatar M. (2018). Teachers’ approaches toward cultural diversity predict diversity-related burnout and self-efficacy. J. Teach. Educ. 69, 408–419. doi: 10.1177/0022487117714244

[ref30] Halgunseth L. C. Peterson A. Stark D. R. Moodie S. (2009). Family engagement, diverse families, and early childhood education programs: An integrated review of the literature [La participación activa de la familia, las familias diversas y los programas de educación de la primera infancia: Análisis integrado de la literatura]. Washington, DC: Pre-K Now and NAEYC.

[ref31] Hayakawa M. Englund M. M. Warner-Richter M. N. Reynolds A. J. (2013). The longitudinal process of early parent involvement on student achievement: a path analysis. NHSA Dialog 16:103. 27867317 PMC5115270

[ref32] Hoover-Dempsey K. V. Sandler H. M. (1997). Why do parents become involved in their children‘s education? Rev. Educ. Res. 67, 3–42. doi: 10.3102/00346543067001003

[ref33] Hoover-Dempsey K. V. Walker J. M. T. Sandler H. M. Whetsel D. Green C. L. Wilkins A. S.. (2005). Why do parents become involved? Research findings and implications. Elem. Sch. J. 106, 105–130. doi: 10.1086/499194

[ref35] Hornby G. Lafaele R. (2011). Barriers to parental involvement in education: an explanatory model. Educ. Rev. 63, 37–52. doi: 10.1080/00131911.2010.488049

[ref36] Hughes J. N. (2010). Identifying quality in preschool education: Progress and challenge. Sch. Psychol. Rev. 39, 48–53. doi: 10.1080/02796015.2010.12087789

[ref37] Hughes V. (2022). Tense times for young migrants: temporality, life-course and immigration status. J. Ethn. Migr. Stud. 48, 192–208. doi: 10.1080/1369183X.2021.1914561

[ref38] Instituto Nacional de Estadísticas and Departamento de Extranjería y Migración. (2022). Available online at: https://serviciomigraciones.cl/estudios-migratorios/estimaciones-de-extranjeros/ (Accessed January 28, 2025).

[ref39] Kalmijn M. (2024). Cultural and social support explanations of the native-migrant gap in the use of day care for pre-school children. J. Ethn. Migr. Stud. 50, 994–1012. doi: 10.1080/1369183X.2023.2245152

[ref40] Kang V. Y. Coba-Rodriguez S. Kim S. (2024). “We need to prepare and adjust”: the school readiness beliefs and practices of Korean families with preschool-aged children. Early Child. Res. Q. 67, 55–66. doi: 10.1016/j.ecresq.2023.11.005

[ref41] Katıtaş S. Coşkun B. Karadas H. (2024). The relationship between teachers’ cultural intelligence and multicultural education attitude: the mediating role of intercultural sensitivity. Int. J. Educ. Res. 127:102443. doi: 10.1016/j.ijer.2024.102443

[ref42] Keles S. Munthe E. Ruud E. (2024). A systematic review of interventions promoting social inclusion of immigrant and ethnic minority preschool children. Int. J. Incl. Educ. 28, 924–939. doi: 10.1080/13603116.2021.1979670

[ref43] Khalfaoui A. García-Carrión R. Villardón-Gallego L. (2020). Bridging the gap: engaging Roma and migrant families in early childhood education through trust-based relationships. Eur. Early Child. Educ. Res. J. 28, 701–711. doi: 10.1080/1350293X.2020.1817241

[ref44] Kim Y. Kang J. (2024). Culturally, linguistically, and legally relevant caring for undocumented immigrant children and their families. J. Early Child. Teach. Educ. 45, 215–231. doi: 10.1080/10901027.2024.2304047

[ref45] Kim J. Yu H. M. (2024). Home-based parent involvement, parental warmth, and kindergarten outcomes among children of immigrant parents. Early Educ. Dev. 35, 343–367. doi: 10.1080/10409289.2022.2153003

[ref46] Kong P. A. Zhang X. Sachdev A. Yu X. (2024). Learning the rules: Chinese immigrant parents’ involvement during their Children’s transition to kindergarten. Early Childhood Educ. J. 52, 681–691. doi: 10.1007/s10643-023-01452-4

[ref47] Lahoz i Ubach S. Cordeu Cuccia C. C. (2021). Sensibilidad intercultural, clima escolar y contacto intergrupal en estudiantes de educación primaria y secundaria de la Región Metropolitana de Santiago de Chile. Rev. Invest. Educ. 39, 131–147. doi: 10.6018/rie.415921

[ref48] Lara L. Saracostti M. (2019). Effect of parental involvement on Children’s academic achievement in Chile. Front. Psychol. 10:1464. doi: 10.3389/fpsyg.2019.01464, PMID: 31316429 PMC6610476

[ref50] Ma X. Shen J. Krenn H. Y. Hu S. Yuan J. (2016). A meta-analysis of the relationship between learning outcomes and parental involvement during early childhood education and early elementary education. Educ. Psychol. Rev. 28, 771–801. doi: 10.1007/s10648-015-9351-1

[ref9003] Martínez-Zelaya G. Chavez-Herting D. Guglielmetti-Serrano F. (2024). Spanish translation, adaptation and psychometric analysis of a short version of the intercultural sensitivity scale in chilean public workers. Psychol. Reports. 00332941241301360. doi: 10.1177/00332941241301360 39542022

[ref51] Martínez-Zelaya G. P. Mera-Lemp M. J. Bilbao M. (2020). Preferencias aculturativas y su relación con la sensibilidad intercultural y el bienestar. Rev. Psicol. 29, 88–102. doi: 10.5354/0719-0581.2020.55961

[ref52] Massing C. Kirova A. Hennig K. (2013). The role of first language facilitators in redefining parent involvement: newcomer families’ funds of knowledge in an intercultural preschool program. J. Childhood Stud. 38, 4–13. doi: 10.18357/jcs.v38i2.15445

[ref53] Mera-Lemp M. J. Bilbao M. Martínez-Zelaya G. Garrido A. (2021). Los estudiantes de carrera en Educación Preescolar ante la inmigración latinoamericana en Chile. Rev. Electrón. Invest. Educ. 23:e23, 1–14. doi: 10.24320/redie.2021.23.e23.3659

[ref54] Mera-Lemp M. J. Pizarro J. J. Guglielmetti-Serrano F. (2025). Bridging cultures: the role of School's cultural diversity climate and cultural sensitivity in immigrant Parents' School involvement. Front. Psychol. 16:1561863. doi: 10.3389/fpsyg.2025.1561863, PMID: 40212317 PMC11984563

[ref55] Mera-Lemp M.-J. Torres-Vallejos J. Guglielmetti-Serrano F. Y. (2024). La amenaza cultural y la actitud de los docentes chilenos hacia la multiculturalidad en la escuela: el rol de la ansiedad exogrupal y la sensibilidad intercultural [Chilean teachers’ cultural threat and attitudes towards multiculturality at school: the role of outgroup anxiety and intercultural sensitivity]. Rev. Española Pedagogía 82, 607–625. doi: 10.22550/2174-0909.4038

[ref56] Ministerio de Relaciones Exteriores de Chile. Reporte de la OCDE sobre Estudiantes Migrantes en la Escuela. Chile en el Exterior. Recuperado 4 de diciembre de. (2024). Available online at: http://www.chile.gob.cl/chile/blog/ocde/reporte-de-la-ocde-sobre-estudiantes-migrantes-en-la-escuela (Accessed December 04, 2024).

[ref57] Murray B. Domina T. Petts A. Renzulli L. Boylan R. (2020). “We’re in this together”: bridging and bonding social capital in elementary school PTOs. Am. Educ. Res. J. 57, 2210–2244. doi: 10.3102/0002831220908848

[ref58] Murray K. W. Finigan-Carr N. Jones V. Copeland-Linder N. Haynie D. L. Cheng T. L. (2014). Barriers and facilitators to school-based parent involvement for parents of urban public middle school students. SAGE Open 4:8030. doi: 10.1177/2158244014558030, PMID: 27088049 PMC4833392

[ref9002] Otero Bracco C. Eisenberg J. (2017). Neighbors link’s parent-child together program: supporting immigrant parents’ integration to promote school readiness among their emergent bilingual children. J Multil. Edu. Res. 7.

[ref60] Özdoğru M. Çevik M. N. Çevik M. S. (2024). Investigation of communication, social intelligence and intercultural sensitivity competencies of teacher candidates in sustainable education by structural equation modeling. Sustain. For. 16:9282. doi: 10.3390/su16219282

[ref61] Pastena A. Sesé A. Trenchs-Parera M. (2024). Impact of plurilingualism and previous intercultural experience on undergraduates’ intercultural sensitivity at the start of university studies. J. Multiling. Multicult. Dev. 45, 1662–1674. doi: 10.1080/01434632.2021.2013854

[ref62] Pérez-Domínguez P. (2024). Evaluation of the intercultural sensitivity according to different variables. Available online at: http://crea.ujaen.es/jspui/handle/10953.1/24864 (Accessed January 28, 2025).

[ref63] Pinto M. Misas M. (2014). La educación inicial y la educación preescolar: Perspectivas de desarrollo en Colombia y su importancia en la con del mundo de los niños. Cult Educ. Soc. 5, 119–140.

[ref64] Reynolds A. D. Crea T. M. Medina J. Degnan E. McRoy R. (2015). A mixed methods case study of parent involvement in an urban high school serving minority students. Urban Educ. 50, 750–775. doi: 10.1177/0042085914534272

[ref65] Rolla A. Rivadeneira M. (2006). ¿Por qué es importante y cómo es una educación preescolar de calidad. Santiago de Chile: Expansiva, Serie En Foco. Available online at: www.expansiva.cl, https://fundacionaprendiz.cl/wp-content/uploads/2020/02/5.-Por-que-es-importante-y-como-es-una-educacion-preescolar-de-calidad.pdf (Accessed January 28, 2025).

[ref66] Romero Díaz F. D. (2024). El contexto sociocultural del niño, como medio para el aprendizaje en la Educación Preescolar. Rev. Neuronum 10:2.

[ref67] Ruiz-Bernardo P. Ribés A. S. Sánchez-Tarazaga L. Mateu-Pérez R. (2024). Intercultural sensitivity and measurement instruments: a systematic review of the literature. Int. J. Intercult. Relat. 102, 102035–102019. doi: 10.1016/j.ijintrel.2024.102035, PMID: 40247951

[ref68] Ryu E.-J. (2022). Predictors of intercultural sensitivity and cultural empathy on multicultural acceptance in nursing students. J. Korean Soc. Integr. Med. 10, 125–134. doi: 10.15268/ksim.2022.10.2.125

[ref69] Şahin B. K. Sak R. Şahin İ. T. (2013). Parents’ views about preschool education. Procedia. Soc. Behav. Sci. 89, 288–292. doi: 10.1016/j.sbspro.2013.08.848

[ref70] Sánchez-Suzuki Colegrove K. (2022). Latinx immigrant parents’ views on learning and collaboration in the early childhood classroom (Opiniones de los padres y las madres latinos inmigrantes sobre el aprendizaje y la colaboración en las aulas de educación preescolar). J. Study Educ. Dev. 45, 678–700. doi: 10.1080/02103702.2022.2059947, PMID: 40101104

[ref71] Sell J. (2017). Storytelling for intercultural understanding and intercultural sensitivity development. En Chlopczyk J. (Ed.), Beyond storytelling: narrative Ansätze und die Arbeit mit Geschichten in Organisationen. Berlin: Springer. 223–249.

[ref72] Servicio Jesuita a Migrantes (2022). Available online at: https://sjmchile.org/incidencia-y-estudios/poblacion-migrante/ (Accessed January 28, 2025).

[ref73] Servicio Nacional de Migraciones (2023). Nueva entrega de Estimación de Población Migrante 2022. Santiago: SERMIG. Available at: https://serviciomigraciones.cl/nueva-estimacion-poblacion-migrante-2022/ (Accessed January 28, 2025).

[ref74] Sirlopú D. Renger D. (2020). Social recognition matters: consequences for school participation and life satisfaction among immigrant students. J. Community Appl. Soc. Psychol. 30, 561–575. doi: 10.1002/casp.2463

[ref75] Sohn S. Wang X. C. (2006). Immigrant parents’ involvement in American schools: perspectives from Korean mothers. Early Childhood Educ. J. 34, 125–132. doi: 10.1007/s10643-006-0070-6

[ref76] Subramaniam L. (2011). Barriers to and facilitators of Latino parent involvement: one Georgia District’s perspective. Statesboro, United States of America: Georgia Southern University. Available at: https://digitalcommons.georgiasouthern.edu/etd/390 (Accessed January 28, 2025).

[ref77] Sylva K. Melhuish E. Sammons P. Siraj I. Taggart B.. (2004). The effective provision of pre-school education (EPPE) project technical paper 12: the final report – effective pre-school education. Available online at: https://dera.ioe.ac.uk/id/eprint/18189/2/SSU-SF-2004-01.pdf

[ref78] Tamam E. Krauss S. E. (2017). Ethnic-related diversity engagement differences in intercultural sensitivity among Malaysian undergraduate students. Int. J. Adolesc. Youth 22, 137–150. doi: 10.1080/02673843.2014.881295

[ref79] Turaşlı N. K. (2016). What is “different”? Intercultural comparison between preschool Children’s perceptions of different people: sample of Poland-Tunisia-Turkey. J. Educ. Soc. Behav. Sci. 13, 1–11. doi: 10.9734/BJESBS/2016/21786

[ref80] Ulrey K. L. Amason P. (2001). Intercultural communication between patients and health care providers: an exploration of intercultural communication effectiveness, cultural sensitivity, stress, and anxiety. J. Health Commun. 13, 449–463. doi: 10.1207/S15327027HC1304_06, PMID: 11771806

[ref81] Valdés Cuervo Á. A. Martín Pavón M. J. Sánchez Escobedo P. A. (2009). Participación de los padres de alumnos de educación primaria en las actividades académicas de sus hijos. Rev. Electr. Invest. Educ. 11, 1–17.

[ref82] Vargas Fonseca I. (2024). La importancia de la educación preescolar. Rev. Neuron. 10, 190–204.

[ref84] Xi C. Wang L. (2024). How parental migration status affects early development of rural children: the indirect role of family socioeconomic status and home environment. Early Educ. Dev. 35, 169–187. doi: 10.1080/10409289.2022.2119798

[ref85] Yamamoto Y. Li J. (2012). What makes a high-quality preschool? Similarities and differences between Chinese immigrant and European American parents’ views. Early Child. Res. Q. 27, 306–315. doi: 10.1016/j.ecresq.2011.09.005

[ref86] Yoshikawa H. Weiland C. Brooks-Gunn J. Burchinal M. R. Espinosa L. M. Gormley W. T.. (2013). Investing in our future: the evidence based on preschool education. Society for Research in Child Development. Available online at: https://eric.ed.gov/?id=ED579818

